# Enhancing Strength-Ductility Synergy in Rolled High-Thermal-Conductivity Mg-Mn-Ce Alloys via Accumulated Strain

**DOI:** 10.3390/ma18204747

**Published:** 2025-10-16

**Authors:** Xu Zhang, Taiki Nakata, Enyu Guo, Wenzhuo Xie, Wenke Wang, Chao Xu, Jing Zuo, Zelin Wu, Kaibo Nie, Xiaojun Wang, Shigeharu Kamado, Lin Geng

**Affiliations:** 1 State Key Laboratory of Precision Welding & Joining of Materials and Structures, Harbin Institute of Technology, Harbin 150001, China; zhangxuhit2023@163.com (X.Z.); zuo_jing@126.com (J.Z.); iszelinwu@163.com (Z.W.); 2 School of Materials Science and Engineering, Harbin Institute of Technology, Harbin 150001, China; xjwang@hit.edu.cn; 3 Department of Mechanical Engineering, Nagaoka University of Technology, Nagaoka 940-2188, Japan; nakata@mech.nagaokaut.ac.jp (T.N.); kamado@mech.nagaokaut.ac.jp (S.K.); 4 School of Materials Science and Engineering, Dalian University of Technology, Dalian 116024, China; eyguo@dlut.edu.cn; 5 School of Materials Science and Engineering, Harbin Institute of Technology, Weihai 264209, China; 22b909125@stu.hit.edu.cn (W.X.); 15b309020@hit.edu.cn (W.W.); 6 College of Materials Science and Engineering, Taiyuan University of Technology, Taiyuan 030024, China; kaibo.nie@gmail.com; 7 Harbin Institute of Technology Suzhou Research Institute, Suzhou 215000, China; genglin@hit.edu.cn

**Keywords:** rolling, second phase, dynamic recrystallization, mechanical properties, thermal conductivity

## Abstract

Magnesium (Mg) alloys are prized as the lightest structural materials but often suffer from a strength–ductility trade-off that is particularly challenging for applications demanding high thermal conductivity. Aiming to resolve this issue, rolled ternary Mg-0.9Mn-1.9Ce (wt.%) alloy sheets were designed and fabricated, and the influence of rolling strain on optimizing the property balance was systematically investigated. The cast alloy was homogenized and rolled to two accumulated strains to obtain S10 (90%) and S20 (95%), followed by microstructure characterization and mechanical/thermal evaluation. Compared with S10, S20 developed finer, more equiaxed grains and a weaker basal texture via enhanced dynamic recrystallization; concurrent fragmentation and uniform dispersion of second-phase particles further contributed to strengthening. Consequently, S20 achieved 14.2% and 13.7% increases in yield and tensile strengths, respectively, with a slight improvement in elongation, while retaining high thermal conductivity (134.4 W·m^−1^·K^−1^ vs. 138.1 W·m^−1^·K^−1^ for S10). The minimal conductivity penalty is attributed to the low solute level in the α-Mg matrix, which limits electron scattering. These results provide experimental and mechanistic guidance for developing rolling Mg alloys that combine high mechanical performance with excellent thermal efficiency.

## 1. Introduction

Magnesium (Mg) alloys are the lightest structural metallic materials, with a density of 1.74 g/cm^3^, offering high specific strength superior to aluminum alloys and excellent recyclability [1,2,3]. The current research direction has shifted towards achieving functionality while maintaining mechanical properties. In particular, the demand for high-thermal-conductivity Mg alloy sheets has been steadily increasing in areas such as electronic packaging, electric vehicles, and advanced thermal management systems [4,5].

Pure magnesium has a relatively high thermal conductivity, second only to copper Cu and Al among industrial metals [6]. However, pure Mg suffers from poor plasticity and low strength at room temperature, primarily due to its hexagonal close-packed (hcp) crystal structure with a limited number of active slip systems and the tendency to form strong basal texture during sheet processing [7,8]. The addition of alloying elements effectively enhances strength and weakens the basal texture, but the issue of the strength-ductility trade-off remains [9,10,11]. Previous studies have shown that solid solution strengthening, dislocation strengthening, and texture strengthening significantly reduce thermal conductivity [12,13,14]. Therefore, achieving a simultaneous enhancement in strength and ductility while maintaining high thermal conductivity in Mg alloys remains a key challenge for their application expansion. The effect of solute atoms on thermal conductivity is an order of magnitude greater than other factors [15]. Designing precipitation-strengthened alloys has been regarded as a potential solution to optimize both mechanical performance and thermal conductivity.

In recent years, Mg-Mn-Ce alloys have become a research hotspot. Zhong et al. [16] found that the addition of Ce refines the grain structure of Mg-0.5Mn alloys in both cast and extruded states and weakens the texture. The ultimate tensile strength (UTS), yield strength (YS), and elongation (EL) of an extruded Mg-0.5Mn-0.3Ce alloy were reported to be 320.9 MPa, 295.9 MPa, and 9.6%, respectively. Zhang et al. [17] reported a Mg-0.9Mn-0.5Ce (wt.%) alloy with a tensile yield strength of 328 MPa and high thermal conductivity (120.8 W·m^−1^·K^−1^) formed by low-temperature extrusion. Liu et al. [5] successfully prepared a Mg-1.5Mn-2.5Ce alloy with fully fragmented second-phase particles via hot extrusion, achieving ultra-high performance with a yield strength of 395.8 MPa and thermal conductivity of 142.1 W·m^−1^·K^−1^. In summary, during melting, Ce refines grains by providing potent heterogeneous nucleation sites and by enhancing constitutional supercooling that suppresses grain coarsening. During extrusion or rolling, abundant second-phase particles initiate a particle-stimulated nucleation (PSN) mechanism to facilitate dynamic recrystallization while simultaneously exerting Zener pinning on grain boundaries. These combined effects stabilize a fine-grained microstructure, thereby enhancing the mechanical strength and thermal conductivity of the alloy. However, most studies on high-strength, high-thermal-conductivity alloys have focused on extruded alloys, with limited research on high-thermal-conductivity alloy sheets with excellent mechanical properties and a lack of studies on related deformation mechanisms and theoretical guidance.

Extrusion and rolling are the two most common deformation techniques for fabricating magnesium alloy sheets. Extrusion, under a triaxial stress state, promotes extensive dynamic recrystallization (DRX) and efficient fragmentation of secondary phases, resulting in significant grain refinement [18]. Rolling, however, operates under a biaxial stress state, where limited shear suppresses particle fragmentation. During inter-pass annealing, recrystallization occurs mainly through recovery-assisted static recrystallization (SRX), in which dynamic recovery reduces dislocation density and organizes subgrains that subsequently transform into recrystallized grains [19,20]. Prolonged annealing may further drive grain growth, affecting the strength–ductility balance. Although extrusion provides notable microstructural advantages, its use in sheet production is constrained by die size and processing efficiency [21]. Rolling offers greater flexibility in controlling sheet dimensions and is therefore considered the most suitable method for large-scale sheet fabrication [22,23].

Xu et al. [24] enhanced the yield strength of a Mg-Gd-Y-Zn-Zr alloy by increasing the accumulated deformation during hot rolling, which promoted precipitation and optimized the spatial distribution and homogeneity of phases. Separately, Jia et al. [25] regulated recrystallization behavior through controlled compressive strain utilizing the PSN mechanism induced by long-period stacking ordered (LPSO) phases. Existing research has mainly focused on texture control and the improvement of dynamic recrystallization (DRX) behavior, but there is a lack of methods for simultaneously enhancing strength and ductility while maintaining high thermal conductivity.

This study addresses the critical challenge of achieving a concurrent improvement in strength and ductility of high-thermal-conductivity magnesium alloys. Different levels of accumulated strain were designed to investigate the coupling mechanism between microstructural evolution and the mechanical–thermal performance of rolled Mg-0.9Mn-1.9Ce (wt.%) alloy sheets. Specifically, the objectives of this work are: (i) to establish a processing strategy that achieves a favorable synergy between strength and ductility while retaining high thermal conductivity; (ii) to elucidate the mechanism by which accumulated strain governs microstructural features and their impact on strength, ductility, and thermal conductivity; and (iii) to provide theoretical and practical guidance for the design of high-thermal-conductivity magnesium sheets with optimized mechanical–thermal balance.

## 2. Materials and Methods

### 2.1. Materials Preparation

The material used in this study was a self-designed Mg-Mn-Ce alloy. The alloy was prepared by metal mold casting at 700 °C using high-purity Mg, Mg-5Mn (wt.%), and Mg-30Ce (wt.%) master alloys. The melting process was carried out in a resistance furnace under a protective atmosphere of CO_2_ + 0.5% SF_6_ to prevent oxidation and burning loss. After stirring, the melt was held at 680 °C for 15 min and then solidified by water cooling. The actual chemical composition, determined at three different positions by inductively coupled plasma optical emission spectroscopy (ICP-OES), was Mg-0.9Mn-1.9Ce (wt.%), designated as ME12 (Table 1). The alloy ingots were cut into cylindrical billets with thicknesses of 10 mm and 20 mm, homogenized at 500 °C for 12 h, and subsequently water-quenched.

The billets were then held at 400 °C for 30 min and rolled using rolls maintained at a surface temperature of 250 °C with a line speed of 5 m/min. The billets with an initial thickness of 10 mm were rolled in seven passes, with thickness reductions of 20%, 30%, 30%, 35%, 40%, 40%, and 40% in each pass, respectively. The final thickness reached 1 mm, corresponding to a cumulative thickness reduction of 90%. Intermediate annealing was performed at 400 °C, with annealing times gradually reduced from 10 min to 5 min (10 min for passes above 5 mm thickness, 5 min for passes below 5 mm). The resulting sheet was designated as S10. The 20 mm billets were rolled in 8 passes to a final thickness of 1 mm, achieving a cumulative strain of 95%. The first pass had a deformation of 20%, passes 2–6 were 30%, and passes 7–8 were 40%. Intermediate annealing was carried out at 400 °C with the same annealing schedule as S10. The resulting sheet was designated as S20. After the final rolling pass, all sheets were subjected to air cooling.

### 2.2. Characterization

Microstructural analysis was conducted using optical microscopy (OM, Olympus BX53M) and field-emission scanning electron microscopy (FE-SEM, TESCAN MAGNA, Brno, s.r.o.) with an Electron Backscatter Diffraction (EBSD) detector of EDAX (California, American). EBSD maps were collected at 20 kV and 10 nA, with a 0.4 μm step size, and analyzed by OIM Analysis™ 8 (EDAX) for crystallographic data. Phase identification was performed via X-ray diffraction (XRD, PANalytical X’Pert PRO, Almelo, Netherlands) with Cu-Kα radiation (λ = 1.5406 Å), scanned over a 2θ range of 20–90° at a speed of 4°/min. Thermodynamic calculations of phase types and fractions were carried out using Pandat™ 2017. OM was observed after chemical etching using an acetic-picral solution, with the composition of 2 g picric acid, 5 mL acetic acid, 5 mL distilled water, and 30 mL ethanol. The second-phase area fraction was quantified by analyzing five representative metallographic images at 200× magnification using Image-Pro Plus 6.0 software.

### 2.3. Mechanical Test and Thermal Conductivity Test

**Figure 1:**
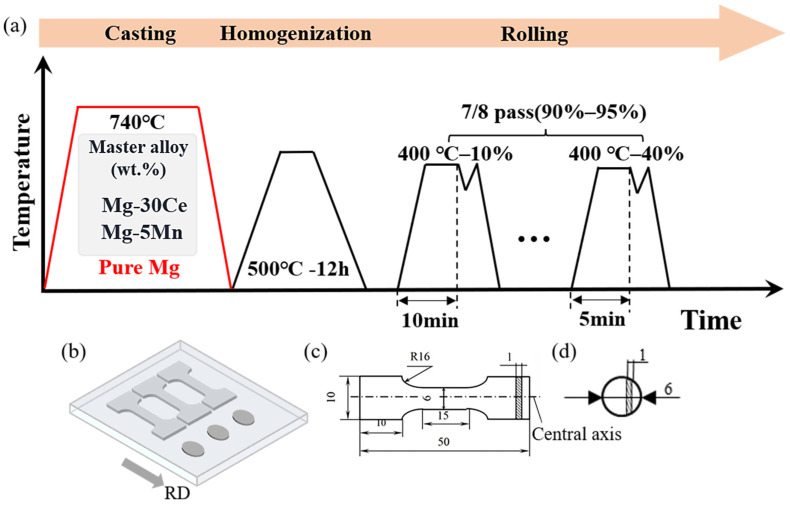
Material preparation process and sample dimensions: (**a**) Schematic of melting, heat treatment, and rolling processes; (**b**) Sampling positions for tensile and thermal conductivity samples; (**c**) Dimensions of room-temperature tensile samples; (**d**) Dimensions of thermal conductivity samples.

Tensile samples were prepared with a gauge length of 15 mm, width of 6 mm, and thickness of 1 mm, and a total length of 50 mm, oriented along the rolling direction (RD). The sample locations are shown in Figure 1b, and the dimensions are shown in Figure 1c. Tensile tests were performed on a Shimadzu Autograph AG-X Plus machine (Kyoto, Japan) at a constant crosshead speed corresponding to an initial strain rate of 10^−3^ s^−1^.

Thermal diffusivity was measured using samples cut from the rolled sheet, with dimensions Φ 6 mm × 1 mm (sample size shown in Figure 1d). Measurements were taken at 25 °C using a Netzsch LFA467 laser flash apparatus (Bavaria, Germany), with three samples measured per condition. Sample density was obtained using the Archimedes method, and the alloy specific heat was calculated using the Neumann-Kopp rule [26]. Thermal conductivity (λ) was calculated using these parameters and the appropriate formula [27].

λ = C_P_ · *α* · *ρ*

where C_p_ (J/(g·K)) is the specific heat capacity, α (mm^2^/s) is the thermal diffusivity, and *ρ* (g/cm^3^) is the density.

## 3. Results

**Figure 2:**
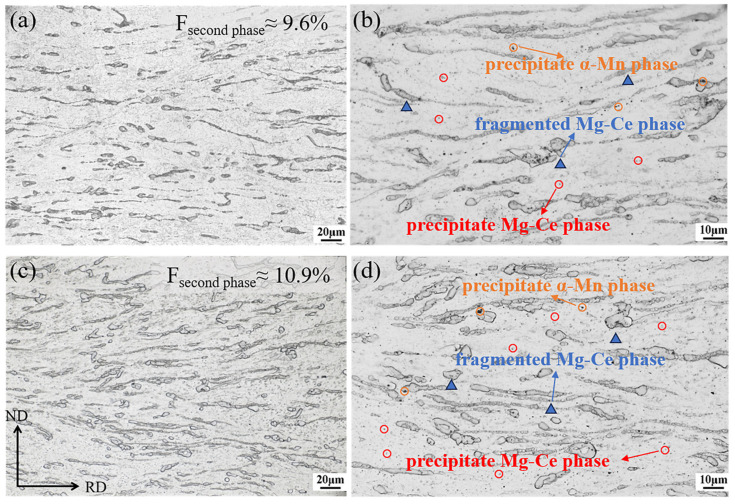
Microstructure of the ME12 rolled alloy: (**a**,**b**) S10; (**c**,**d**) S20.

Figure 2 presents the microstructural morphology of the ME12 alloy subjected to 90% and 95% rolling reductions. The area fractions of secondary phases were comparable, with approximately 9.6% in S10 as shown in Figure 2a and 10.9% in S20 as shown in Figure 2c, while the larger cumulative deformation in S20 promoted more effective fragmentation of the secondary phases. These phases were distributed discontinuously along the RD in banded arrangements and mainly exhibited elongated morphologies. In S10, coarse particles of about 5 μm coexisted with finer ones of 1–2 μm. As illustrated in Figure 2b,d, the blue triangles denote fragmented Mg-Ce phases, the yellow circles indicate α-Mn precipitates, and the red circles highlight Mg-Ce precipitates. Although the types of precipitates were similar in both samples, the S20 sample exhibited a higher number density, suggesting a stronger tendency for dynamic precipitation. This phenomenon will be further discussed in the following section.

**Figure 3:**
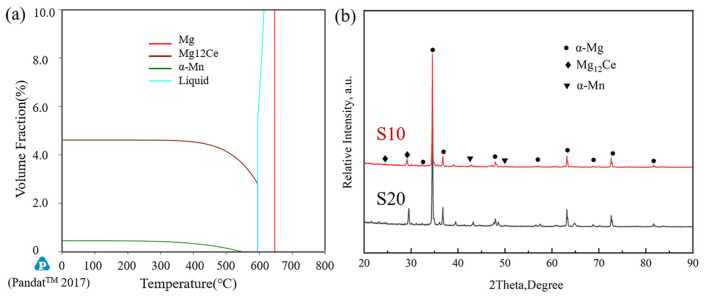
Phase analysis of the ME12 alloy: (**a**) Second-phase content calculation diagram; (**b**) XRD diffraction spectrum.

To determine the phase composition and fractions in the ME12 alloy, phase evolution with temperature was simulated using the CALPHAD method in Pandat software, as shown in Figure 3a. The results indicate that, in addition to the primary α-Mg matrix, two secondary phases, Mg_12_Ce and α-Mn, are present. The volume fraction of the Mg_12_Ce phase was approximately 4.6%, and that of the α-Mn phase was about 0.5%. The Mg_12_Ce phase started to precipitate at higher temperatures and remained stable over a wide temperature range, while the α-Mn phase had a narrower stability range and a lower volume fraction. The liquid phase completely disappeared at around 630 °C, which corresponded to the alloy’s solidification endpoint. To verify the thermodynamic predictions, XRD analysis of the as-cast alloy was performed (Figure 3b). The results confirmed the presence of α-Mg, Mg_12_Ce, and α-Mn phases, consistent with the calculated results. The Mg_12_Ce diffraction peaks were relatively strong, indicating a higher content of rare-earth intermetallic compounds in the alloy.

**Figure 4:**
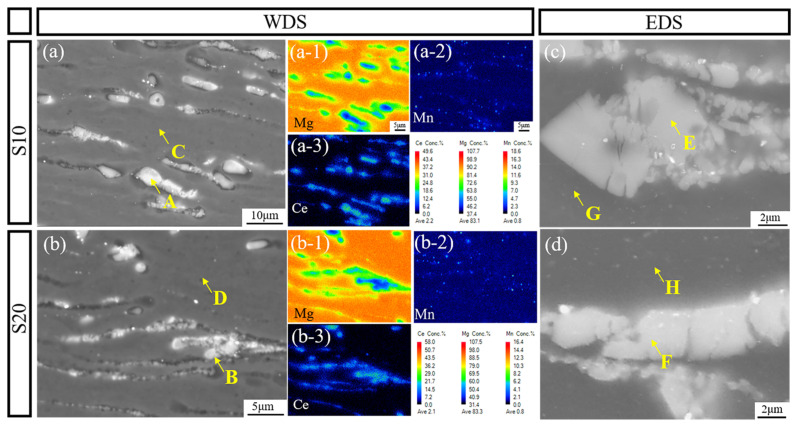
EPMA-WDS and SEM-EDS analysis of the ME12 alloy, S10: (**a**) BSE images; (**a-1**) Mg-mapping; (**a-2**) Mn-mapping; (**a-3**) Ce-mapping; (**c**) EDS point analysis positions; S20: (**b**) BSE images; (**b-1**) Mg-mapping; (**b-2**) Mn-mapping; (**b-3**) Ce-mapping; (**d**) EDS point analysis positions.

Figure 4 illustrates the point and mapping analyses performed by EPMA-WDS (Electron Probe Microanalyzer with Wavelength Dispersive Spectroscopy), together with the EDS (Energy Dispersive Spectroscopy) point analysis locations, for the S10 and S20 samples. Figure 4a-1–a-3 present the WDS elemental distribution maps of Mg, Mn, and Ce in the S10 sample, while Figure 4b,b-1–b-3 correspond to the S20 sample. The results demonstrate that Ce and Mn were significantly enriched in the second-phase regions, with Ce exhibiting more pronounced segregation. Mn was mainly distributed around the Mg–Ce phases, appearing as fine bright particles, and after rolling, both Ce-rich and Mn-rich phases were aligned along the RD. The WDS mapping revealed a similar distribution pattern in both the S10 and S20 samples. To further quantify the compositional differences between the matrix and the second phases, WDS point analyses were conducted at sites A–D, and EDS point analyses were carried out at sites E–H.

Table 2 summarizes the WDS and EDS point analysis results for the S10 and S20 samples. WDS analyses at points A and B corresponded to second-phase particles, where the Ce content was relatively high (approximately 5 at.%), with similar values in both samples. Points C and D represented the α-Mg matrix, where the Ce and Mn contents were extremely low (Ce ≤ 0.1 at.% and Mn ≤ 0.3 at.%). In contrast, EDS point analyses at points E and F further confirmed Ce enrichment in the second-phase particles, while points G and H indicated that the solute content in the matrix remained very low, consistent with the WDS results. This consistent microstructural feature between S10 and S20 suggests that the alloy design and thermomechanical processing promoted sufficient precipitation of alloying elements, thereby leaving only trace solute atoms in the matrix.

**Figure 5:**
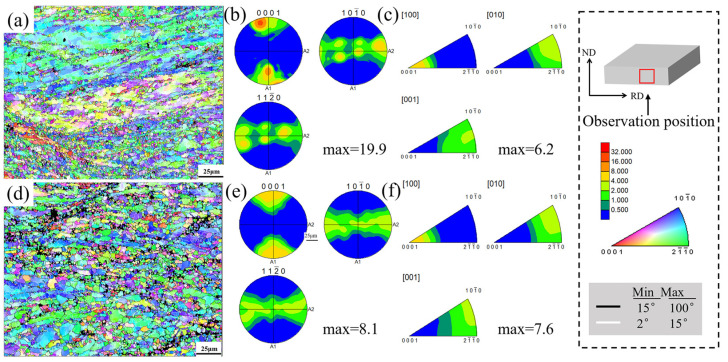
Orientation analysis of the ME12 rolled alloy, S10: (**a**) IPF map; (**b**) Pole figure; (**c**) Inverse pole figure; S20: (**d**) IPF map; (**e**) Pole figure; (**f**) Inverse pole figure.

Figure 5 compares the IPF (Inverse Pole Figure) map and pole figure textures of ME12 alloy at different cumulative rolling reductions. As shown in Figure 5a, in the S10 sample, the grains still exhibited a distinct elongation along the rolling direction, with two large blocks of deformed grains present in the upper and middle sections, retaining clear characteristics of cast grain structure. The majority of the orientations remained consistent, while a small portion of the regions underwent dynamic recrystallization and exhibited subgrains with small-angle grain boundaries, resulting in partial orientation deviation. As shown in Figure 5b, the pole figures of the S10 sample revealed strong orientation concentration in the (0001) and {10$\bar{1}$0} planes, with the maximum orientation density of 19.9 mrd.

In contrast, the S20 sample exhibited a large area of fine, equiaxed recrystallized grains, indicating that higher accumulated strain significantly promoted dynamic recrystallization. Correspondingly, the texture, originally dominated by a strong, oriented rolling basal texture, gradually dispersed. The concentration in the S20 sample was significantly reduced (maximum 8.1 mrd), and the inverse pole figure similarly showed a shift toward randomization.

**Figure 6:**
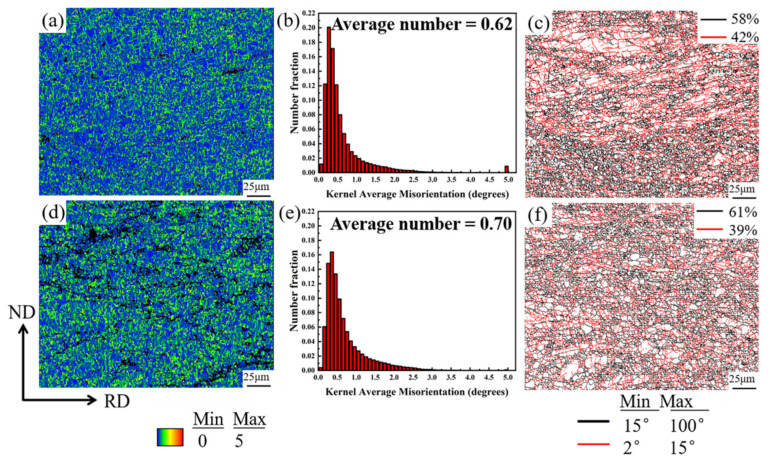
ME12 alloy S10 sample: (**a**) KAM map; (**b**) KAM value distribution; (**c**) Grain boundary distribution; and S20 sample: (**d**) KAM map; (**e**) KAM value distribution; (**f**) Grain size distribution.

Figure 6 compares the Kernel Average Misorientation (KAM), grain boundary misorientation characteristics, and grain size distribution evolution of ME12 alloy under different cumulative rolling strains (90% and 95%). In Figure 6a,b, the KAM maps of the S10 sample show predominantly low values (blue/green), with the histogram indicating an average intrinsic distortion of approximately 0.62°. In contrast, for the S20 sample, after further accumulated strain, the overall KAM level increased slightly to an average of 0.70°. Fine grains and numerous grain boundaries still show local strain gradients, which caused a slight increase in the statistical average KAM. The grain boundary distribution maps in Figure 6c,f reveal that the network of high-angle grain boundaries (HAGBs, >15°) in S20 is more continuous, indicating that the accumulated strain promoted subgrain rotation and transformation into high-angle grain boundaries, thus enhancing dynamic recrystallization. From S10 to S20, the fraction of LAGBs decreased from 42% to 39%, while the fraction of HAGBs increased from 58% to 61%, indicating that the accumulated strain promoted subgrain rotation and transformation into high-angle grain boundaries, thereby enhancing dynamic recrystallization.

**Figure 7:**
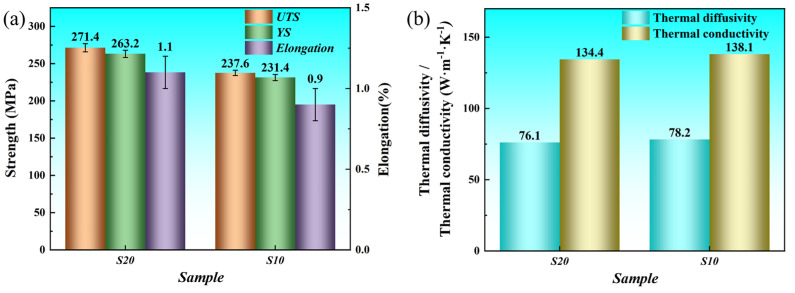
ME12 alloy S10 and S20 samples: (**a**) Mechanical properties; (**b**) Thermal conductivity.

Figure 7 illustrates the differences in mechanical and thermal properties between the S10 and S20 samples. The UTS and YS of S20 were significantly higher than those of S10, increasing by 14.2% and 13.7%, respectively, with values of approximately 271.4 MPa and 263.2 MPa for S20 compared to 237.6 MPa and 231.4 MPa for S10. While the strength of S20 was enhanced, its elongation of 1.1% remained comparable to or slightly superior to that of S10, which had an elongation of 0.9%. The thermal diffusivity and thermal conductivity of S20 were slightly lower than those of S10, with values of 76.1 and 134.4 W·m^−1^·K^−1^ for S20 compared to 78.2 and 138.1 W·m^−1^·K^−1^ for S10. The decrease in thermal conductivity was only 2.7%, with S20 still maintaining approximately twice the conductivity of AZ61 [28]. The low solute content in the α-Mg matrix is the key factor contributing to the excellent thermal conductivity observed in both S10 and S20. The extremely low solute content in the α-Mg matrix effectively reduces electron scattering, ensuring high thermal conductivity. Moreover, the effective segregation of solute elements into dispersed second-phase particles further reduces their concentration in the matrix, preserving the intrinsic high thermal conductivity of pure Mg.

As shown in Table 3, compared with common Mg alloys in terms of mechanical and thermal properties, the rolled Mg-Mn-Ce alloy developed in this study exhibits a clear advantage of achieving enhanced yield strength and elongation while retaining a high thermal conductivity, although its ductility is still relatively limited. This work provides both experimental data and theoretical analysis for the development of high-strength and high-thermal-conductivity Mg alloy sheets, thereby complementing the current research that has been predominantly focused on extruded alloys.

## 4. Discussion

### 4.1. Effect of Accumulated Strain on Strength

**Figure 8:**
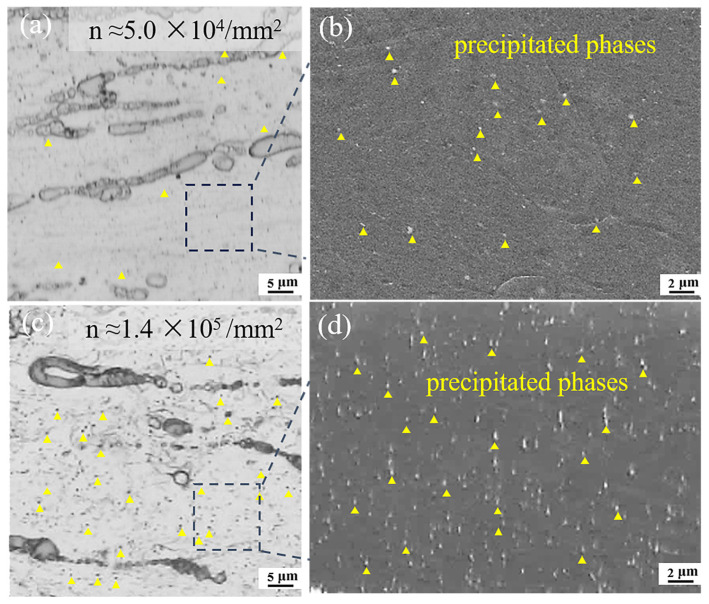
Precipitate phase observation of ME12 alloy: (**a**) S10-OM image; (**b**) S10-SEM image; (**c**) S20-OM image; (**d**) S20-SEM image.

Figure 8 and Table 4 compare the distribution characteristics and number density of second-phase particles in the S10 and S20 samples. The second phase number density in S10 was relatively low (~5.0 × 10^4^/mm^2^). In contrast, the number density in S20 was significantly higher (~1.4 × 10^5^/mm^2^), with particles more densely and uniformly distributed. This difference arises from the larger initial billet thickness and higher accumulated strain in S20, which provide a higher volume fraction of the second phase and promote shear fragmentation during rolling. Additionally, the longer deformation path in S20 results in greater plastic work accumulation. Shear stresses drive the fragmentation of second-phase particles, further promoting particle refinement and homogenization [34,35]. In contrast, S10, with lower accumulated strain and thinner initial billets, exhibits weaker particle fragmentation, resulting in a much sparser distribution of second-phase particles. These results indicate that the coupling of initial billet geometry and accumulated strain plays a crucial role in the evolution of second-phase particles during the rolling process of Mg-Mn-Ce alloys.

**Figure 9:**
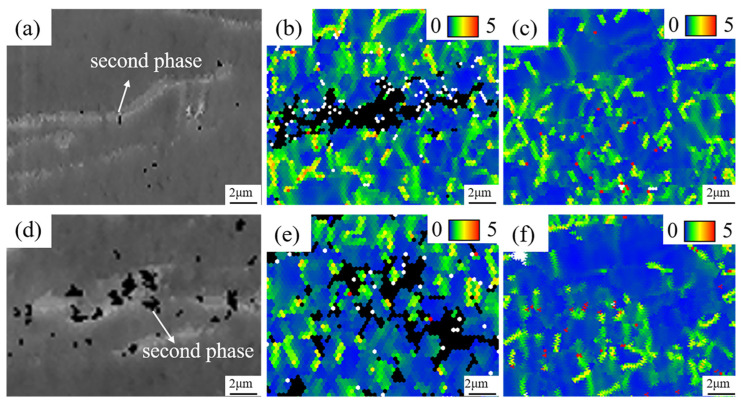
Interaction between second phase and dislocations in ME12 alloy: S10: (**a**) Second-phase region SEM; (**b**) Second-phase region KAM map; (**c**) Matrix region KAM map; S20: (**d**) Second-phase region SEM; (**e**) Second-phase region KAM map; (**f**) Matrix region KAM map.

Figure 9 shows the SEM images and corresponding KAM maps of the second-phase aggregation regions in the ME12 alloy under different rolling conditions. Figure 9a,d display the distribution of second-phase particles, with the black particles representing unidentifiable regions of the second phase. Figure 9b,e show the KAM maps of regions containing second-phase particles, revealing a significant increase in local misorientation, which indicated a higher dislocation density in these regions. The black particles corresponded to the second-phase particles at the same location. In contrast, Figure 9c,f show regions without second-phase particles, where the KAM values were generally low, primarily in the blue region, indicating lower dislocation density [20]. This dislocation density difference suggests a strong interaction between the second phase and dislocations during deformation. The hard and thermally stable Mg_12_Ce and α-Mn particles act as obstacles to dislocation motion, causing dislocations to accumulate around them, forming localized high-strain regions that promote dislocation multiplication and storage [36].

In the S20 sample, the second-phase particles were more densely distributed and smaller in size, indicating that higher rolling deformation promoted particle fracture and redistribution. Additionally, under high accumulated strain, these particles (larger than 1 μm) induced dynamic recrystallization through the PSN mechanism [37]. Furthermore, as shown in Figure 2, with approximately 10.9% second-phase area fraction in the S20 sample, the second phase significantly enhanced its ability to impede dislocation motion during deformation. The large number of uniformly distributed second-phase particles promotes extensive dislocation accumulation at the particle-matrix interfaces, intensifying local strain concentration, inhibiting dislocation slip, and effectively increasing the critical shear stress required for further plastic deformation [38,39]. As a result, the S20 sample demonstrates a significant increase in yield strength and tensile strength, exhibiting a deformation-induced second phase strengthening mechanism that synergistically enhances the microstructural evolution.

### 4.2. Effect of Accumulated Strain on Plasticity

**Figure 10:**
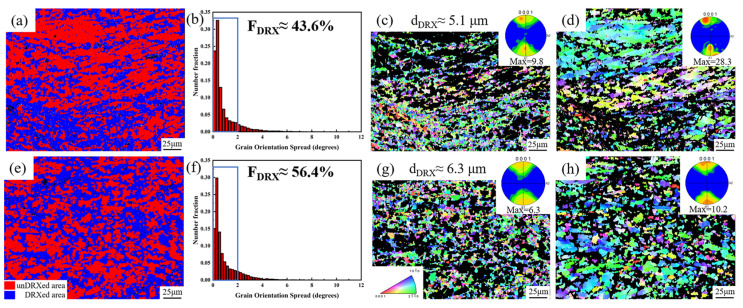
Recrystallization microstructure differentiation in ME12 alloy: S10: (**a**) GOS map; (**b**) GOS distribution histogram; (**c**) Recrystallized region IPF; (**d**) Unrecrystallized region IPF; S20: (**e**) GOS map; (**f**) GOS distribution histogram; (**g**) Recrystallized region IPF; (**h**) Unrecrystallized region IPF.

The DRX behavior of the ME12 alloy is significantly influenced by the accumulated strain during rolling. By separating recrystallized and unrecrystallized regions using a GOS threshold of 2° [3], it was found that the texture intensity in the recrystallized regions was markedly weaker than that in the unrecrystallized regions. As shown in Figure 10a,b,e,f, the recrystallized fraction of the S20 sample (~56.4%) was higher than that of S10 (~43.6%). Moreover, in Figure 10c, the recrystallized grain size of the S10 sample (5.1 μm) was slightly smaller than that of the S20 sample (6.3 μm) shown in Figure 10g, indicating that increasing the rolling reduction from 90% to 95% effectively promoted DRX. In the S10 sample, the pole figure of the recrystallized region exhibited a maximum pole density of 9.8, whereas the unrecrystallized regions showed elongated grains along the rolling direction and a stronger basal texture (maximum texture intensity of 28.3). In contrast, the S20 sample exhibited a weaker basal texture (maximum intensity of 10.2), and the maximum pole density of its recrystallized region (6.3) was also significantly lower than that of S10. These results indicate that the more pronounced DRX in S20 generated randomly oriented recrystallized grains, thereby contributing to texture weakening.

The difference in DRX behavior can be attributed to the higher number density and more uniform distribution of fine second-phase particles in S20. These particles act as effective nucleation sites for PSN, promoting DRX under high-strain conditions. Furthermore, the dense distribution of particles in S20 enhances local strain concentration at the particle–matrix interfaces, promoting dislocation accumulation and new grain nucleation [40]. In parallel, during subsequent annealing after rolling, dynamic recovery (DRV) also plays an important role. By facilitating dislocation annihilation and subgrain boundary formation, DRV not only reduces the stored energy but also provides favorable conditions for subsequent DRX, making it an important pathway for promoting recrystallization in rolled sheets. Therefore, the higher accumulated strain, enhanced DRV activity, and refined second-phase distribution in S20 lead to a higher DRX fraction, smaller recrystallized grains, and a weaker basal texture.

**Figure 11:**
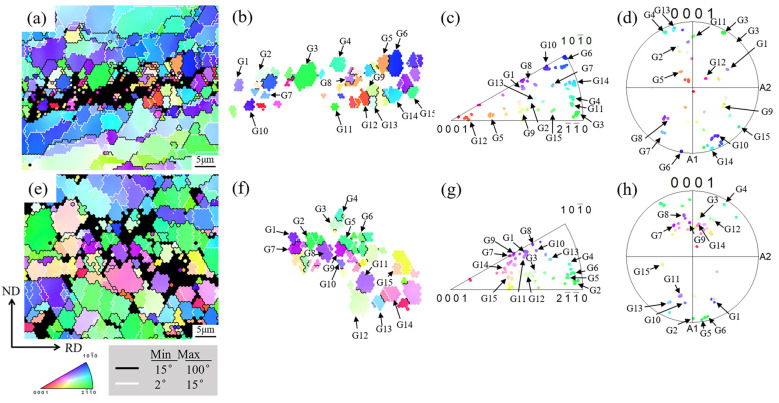
Grain orientation analysis of specific regions in ME12 alloy: S10: (**a**) IPF map; (**b**) Recrystallized grain IPF map; (**c**) Recrystallized grain inverse pole figure; (**d**) Recrystallized grain pole figure; S20: (**e**) IPF map; (**f**) Recrystallized grain IPF map; (**g**) Recrystallized grain inverse pole figure; (**h**) Recrystallized grain pole figure.

Figure 11 highlights the grain orientation analysis in specific regions of the ME12 alloy, demonstrating the significant role of the PSN mechanism in the DRX evolution of ME12 alloy under different rolling conditions. In both S10 and S20 samples, fine recrystallized grains exhibited discontinuous distribution around second-phase particles (black regions in Figure 11a,e), which was a typical feature of the PSN mechanism. The grains labeled G1-G15 (Figure 11b,f) represented typical recrystallized grains near the second phase, used for further orientation analysis. The orientation distribution of these grains was visualized in the {0001} pole figures and inverse pole figures (Figure 11c,d for S10, and Figure 11g,h for S20). In S10, grains such as G1, G3, G5, G8, and G11 showed a relatively dispersed orientation distribution, but there was still a tendency for clustering toward the basal plane, indicating that some local texture was retained. In contrast, grains such as G2, G4, G6, G9, and G14 in S20 exhibited a broader orientation distribution, with no prominent texture components, indicating a higher degree of orientation randomization [41].

This behavior suggests that PSN-induced recrystallized grains nucleate at different orientations due to the local strain heterogeneity around the particles. In S20, the second-phase particles are more thoroughly fragmented and more uniformly distributed, which promotes more frequent and dispersed PSN events, resulting in a higher proportion of weak-texture recrystallized grains [42]. Therefore, compared to S10, the orientation distribution near the particles in S20 is more random, and the texture strength is lower. These results emphasize that the PSN mechanism not only significantly refines the grain size but also effectively weakens the basal texture by randomizing the orientation of new grains, especially when higher accumulated strain and a more uniform distribution of second-phase particles are achieved, improving the alloy’s plasticity [43].

**Figure 12:**
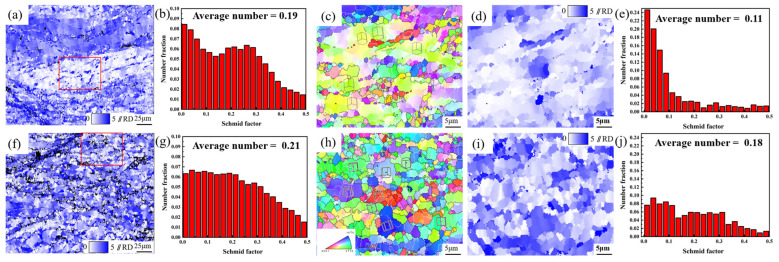
ME12 alloy S10 sample: (**a**) SF map; (**b**) SF distribution; (**c**) IPF map (red-boxed area); (**d**) SF map (red-boxed area); (**e**) SF distribution (red-boxed area); S20 sample: (**f**) SF map; (**g**) SF distribution; (**h**) IPF map (red-boxed area); (**i**) SF map (red-boxed area); (**j**) SF distribution (red-boxed area).

Figure 12 shows the overall and selected area Schmid factor (SF) distributions of the deformed grains in the ME12 alloy under different rolling conditions. In the S10 sample (Figure 12a), large clusters of deformed grains exhibited similar orientations, particularly evident in the magnified area of Figure 12c. The average SF of these grains was low (~0.19), indicating a poor ability to activate the required slip systems, especially the basal slip system. This could lead to uneven strain distribution, promoting strain hardening and negatively impacting the uniformity of ductility and plastic flow.

In contrast, the S20 sample (Figure 12f) showed a more diverse grain orientation distribution, with fewer large clusters of similarly oriented grains. However, the average SF remained low (~0.21), slightly higher than that in S10. The magnified area in Figure 12h revealed that despite more varied grain orientations, some regions with large grains still exhibited low SFs. As shown in Figure 12e, these regions had SFs as low as 0.11, further exacerbating the issue of uneven deformation. Grains with low SFs are prone to localized plastic deformation, which not only limits strain uniformity but also promotes the formation of strain gradients, increasing the risk of microstructural instability and crack propagation [44].

**Figure 13:**
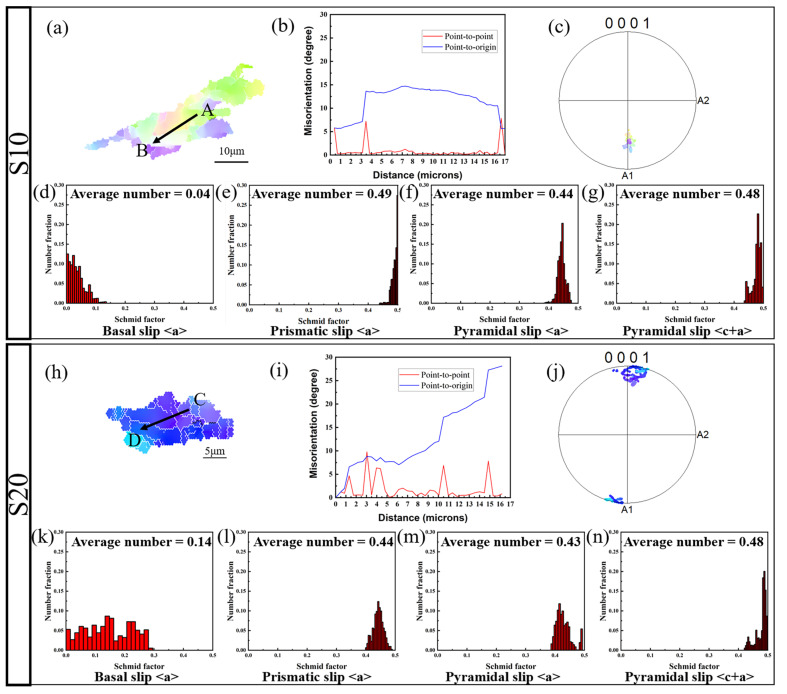
Deformed grain characterization analysis of ME12 alloy: S10: (**a**) Deformed grain IPF map; (**b**) Misorientation-distance map; (**c**) Pole figure; (**d**–**g**) SF distribution histograms for various slip systems. S20 sample: (**h**) Deformed grain IPF map; (**i**) Misorientation-distance map; (**j**) Pole figure; (**k**–**n**) SF distribution histograms for various slip systems.

Figure 13 compares the deformation behavior of S10 (a–g) and S20 (h–n) samples, focusing on the differences in grain orientation, pole figure textures, and the SF distributions of the main slip systems. In the S10 sample, the deformed grains analyzed along the AB line (Figure 13a) exhibit a significant orientation gradient (Figure 13b), reflecting strong shear deformation within the grains. The corresponding pole figure (Figure 13c) shows orientation clustering near the basal pole, indicating a strong texture. The SF distribution (Figure 13d–g) reveals that the average SF for the basal <a> slip system is very low (~0.04), suggesting that basal slip is difficult to activate. In contrast, the SFs for the prismatic <a> and pyramidal <c + a> slip systems are higher (~0.49 and ~0.48), indicating that non-basal slip may dominate plastic deformation in S10 when basal slip is restricted [45]. In the S20 sample, the deformed grains analyzed along the CD line (Figure 13h) show a lower orientation gradient (Figure 13i), indicating reduced shear deformation within the grains. The pole figure (Figure 13j) shows weakened texture strength, suggesting that dynamic recrystallization has enhanced and caused some degree of orientation randomization. The SF distribution (Figure 13k–n) shows that the average SF for the basal slip system has increased (~0.14), while the SFs for the prismatic <a> and pyramidal <c + a> slip systems remain high (~0.44–0.48), indicating a more balanced slip system activity in S20 [46].

In summary, in S10, due to unfavorable basal slip conditions, plastic deformation primarily relies on non-basal slip, leading to an increased orientation gradient and the formation of localized shear bands. In contrast, S20, through grain refinement and orientation randomization, alleviates local constraints and promotes the cooperative activation of multiple slip systems, resulting in a more uniform distribution of strain and improved plasticity coordination.

**Figure 14:**
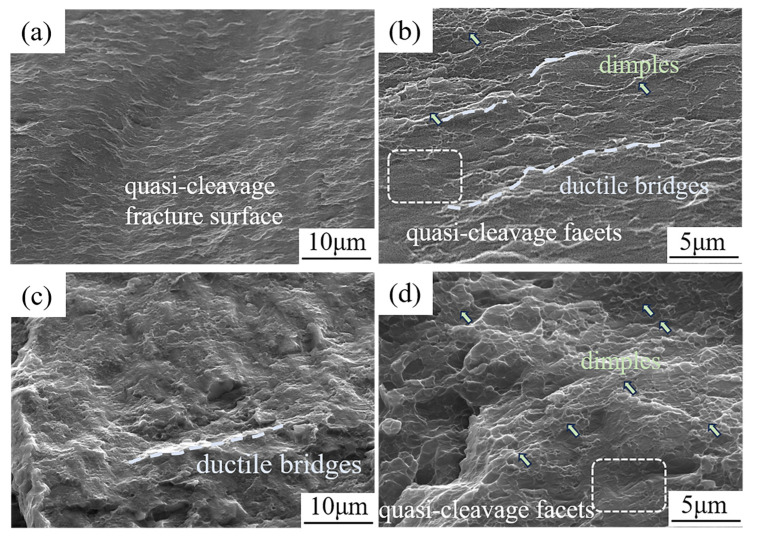
SEM fracture morphologies of the ME12 alloy after tensile testing. S10 sample: (**a**) low-magnification image; (**b**) high-magnification image. S20 sample: (**c**) low-magnification image; (**d**) high-magnification image.

Figure 14 shows the tensile fractography of S10 and S20. S10 (Figure 14a,b) is quasi-cleavage–dominated, with small quasi-cleavage facets intersected by shallow ductile bridges; only a few tiny, nearly flat dimples are present, indicating limited plasticity. In contrast, S20 (Figure 14c,d) displays a surface mainly covered by very shallow/flat dimples with local quasi-cleavage; the dimples are larger and more numerous, evidencing greater plastic accommodation and crack-tip blunting. This evolution—from “quasi-cleavage with ductile bridges” (S10) to “shallow dimpled fracture with local quasi-cleavage” (S20)—is consistent with the higher elongation of S20.

## 5. Conclusions

This study reveals the coupling mechanism of accumulated strain control on the microstructure evolution and mechanical–thermal performance of Mg-0.9Mn-1.9Ce (wt.%) alloys, providing both experimental validation and mechanistic insights for balancing strength, ductility, and thermal conductivity.

(1) By controlling the accumulated strain, the strength–ductility synergy of Mg-Mn-Ce alloys was effectively improved. In the S20 sample with 95% deformation, UTS and YS increased by 14.2% and 13.7%, respectively, while elongation rose slightly, demonstrating that higher accumulated strain can break the conventional strength–ductility trade-off.
(2) Both S10 and S20 samples maintained excellent thermal conductivity (138.1 and 134.4 W·m^−1^·K^−1^), primarily due to the low solute content in the α-Mg matrix, which minimized electron scattering. Moreover, accumulated strain promoted dynamic precipitation, which alleviated the negative effect of dislocation density on thermal transport, thereby sustaining high conductivity.
(3) The increase in accumulated strain and billet thickness enhanced the interaction between second-phase particles and dislocations, raising dislocation density and refining grains. Particle-stimulated nucleation further induced recrystallized grains with random orientations, weakened the basal texture, and activated non-basal slip systems, thus improving plasticity and deformation uniformity.
(4) The S20 sample with higher accumulated deformation exhibited mixed fracture features with tear ridges and increased surface roughness, indicating greater plastic deformation capacity, enhanced crack propagation resistance, and superior ductility.

## Figures and Tables

**Table 1:** ICP element content measurement results.

ICP	Nominal Composition (wt.%)	Mn	Ce	Mg
1	Mg-1.0Mn-2.0Ce (ME12)	1.0	2.0	Bal.
2		0.8	1.8	Bal.
3		0.9	1.9	Bal.

**Table 2:** EDS and WDS points scan element content percentages (at.%).

Points	Analysis Type	Element (at.%)		
		Mg	Mn	Ce
A	WDS	94.4	0.5	5.1
B		94.2	0.5	5.3
C		99.6	0.3	0.1
D		99.6	0.3	0.1
E	EDS	94.7	0.4	4.9
F		95.1	0.5	4.4
G		99.5	0.4	0.1
H		99.6	0.3	0.1

Note: Points A–D were analyzed by WDS, while points E–H were analyzed by EDS.

**Table 3:** Comparison of the mechanical and thermal properties between the present work and common Mg alloys.

Alloy Composition (at%)	Condition	Mechanical Properties		Thermal Conductivity	Reference
		σ 0.2 (MPa)	E (%)	TC (W·m^−1^·K^−1^)	
Mg-0.9Mn-1.9Ce	As-rolled (ε = 95%)	237.6	1.1	138.1	Present work
Mg-0.9Mn-1.9Ce	As-rolled (ε = 90%)	263.2	0.9	134.4	Present work
Mg-3Al-1Zn	As-rolled	175	16	76	[29]
Mg-5.0Al-3.0Ca	As-extruded	337	5.2	118	[30]
Mg-5Zn-1Mn	As-extruded	268	14.8	122	[15]
Mg-4Zn-0.4Zr	As-extruded	255	7	126	[15]
Mg-0.9Mn-0.5Ce	As-extruded	285	11.3	113	[31]
Mg−2.6Al−4.2La	As-extruded	180	9.5	125	[32]
Mg-11Y-5Gd-2Zn-0.5Zr	As-cast	-	-	23	[33]

**Table 4:** Statistical number density of nano-precipitates in ME12 alloy.

Sample	Statistical Range (μm)	Number Density (Count/mm^2^)
S10	100 × 100	5.0 × 10^4^
S20	100 × 100	1.4 × 10^5^

## Data Availability

The original contributions presented in this study are included in the article. Further inquiries can be directed to the corresponding author.
